# Think Adnexal Tumor Beyond the Usual Site: Fine-Needle Aspiration Cytology of Trichoblastoma Presenting as a Large Subcutaneous Mass in the Thigh

**DOI:** 10.3390/diagnostics16101483

**Published:** 2026-05-13

**Authors:** Hidetoshi Satomi, Ayumi Ryu, Azusa Shingetsu, Satoshi Tanada, Keiichiro Honma

**Affiliations:** 1 Department of Diagnostic Pathology and Cytology, Osaka International Cancer Institute, Osaka 541-8567, Japan; 2 Department of Clinical Laboratory, Osaka International Cancer Institute, Osaka 541-8567, Japan; 3 Department of Pathology, Osaka General Medical Center, Osaka 558-0001, Japan

**Keywords:** fine-needle aspiration cytology, trichoblastoma, extra-craniofacial, cytomorphology, differential diagnosis, immunohistochemistry, basal cell carcinoma, follicular adnexal tumor

## Abstract

**Background/Objectives**: Trichoblastoma is a benign follicular adnexal tumor that typically arises on the head and neck. Large variants at atypical locations pose considerable diagnostic challenges because their clinical presentation can be indistinguishable from malignant soft tissue neoplasms. Herein, we describe a case of trichoblastoma presenting as a large subcutaneous thigh mass that was correctly diagnosed by fine-needle aspiration cytology. **Case Presentation:** A 49-year-old male presented with a 7 cm, slowly enlarging, subcutaneous mass in the left thigh of 20 years’ duration. Magnetic resonance imaging raised the possibility of a low-grade sarcoma. Fine-needle aspiration cytology yielded cohesive clusters of basaloid cells with peripheral palisading, delicate spindle-shaped follicular stromal cells intimately admixed with the epithelial component, and orangeophilic keratinous material in the background. The absence of nuclear atypia, mitotic figures, and mucinous stroma supported a preoperative cytological diagnosis of a benign follicular germinative tumor consistent with trichoblastoma, thereby guiding conservative surgical excision. Histopathological examination confirmed the diagnosis. Immunohistochemistry revealed focally positive BerEP4, CD34-positive stroma, negative androgen receptor, and positive bcl-2, consistent with trichoblastoma and distinguishing the tumor from basal cell carcinoma. The patient remained recurrence-free 12 months after surgery. **Conclusions:** Careful assessment of characteristic cytomorphological features, particularly a dual population of basaloid epithelial cells with peripheral palisading and specialized follicular stromal cells, is vital for the accurate preoperative cytological characterization of trichoblastoma, even at atypical anatomical sites.

## 1. Introduction

Trichoblastoma is a benign cutaneous adnexal tumor exhibiting follicular germinative differentiation, typically presenting as a slowly growing, well-circumscribed nodule on the head and neck [1,2]. Large trichoblastomas exceeding 5 cm are uncommon [3], and presentation as a large subcutaneous mass on the extremities is exceedingly rare. In such cases, clinical and imaging features typically lead to a differential diagnosis of soft tissue neoplasms, and the follicular nature of the lesion may not be suspected preoperatively.

Fine-needle aspiration (FNA) cytology can provide important preoperative information to guide surgical planning. Descriptions of the cytological features of trichoblastoma are limited, and predominantly involve lesions at typical locations on the face, head and neck [1,4,5]. Trichoblastoma yields cohesive clusters of bland basaloid cells with peripheral palisading and specialized follicular mesenchymal cells; these features may aid the recognition of the follicular nature of the tumor on cytological specimens. However, no previous reports have described these cytological features in lesions at atypical locations, where clinical suspicion for trichoblastoma is typically low. To our knowledge, no previous report has provided a detailed cytological description of trichoblastoma diagnosed by FNA at an extra-craniofacial site.

Accurate preoperative cytological characterization is clinically meaningful because it can guide the extent of surgery; a benign follicular tumor may be managed by conservative local excision, whereas a suspected sarcoma typically necessitates wider resection with correspondingly greater morbidity. Herein, we report a case of trichoblastoma presenting as a large subcutaneous mass in the thigh, in which careful analysis of the features revealed by FNA cytology enabled the correct preoperative diagnosis.

## 2. Detailed Case Description

A 49-year-old man presented for evaluation of a slowly enlarging subcutaneous mass in the left thigh of approximately 20 years’ duration. The lesion had recently increased in size, prompting clinical referral. Physical examination revealed a firm, mobile, non-tender subcutaneous nodule measuring approximately 7 cm in the largest dimension. No regional lymphadenopathy was detected. The overlying skin was intact and unremarkable.

**Figure 1:**
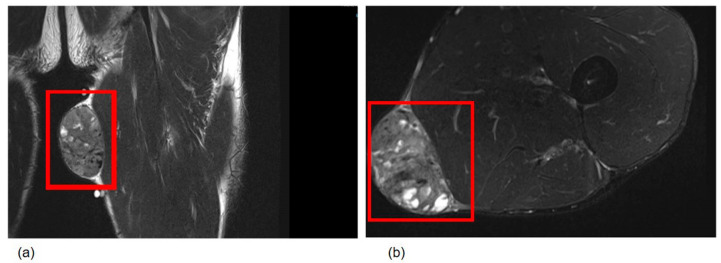
Magnetic resonance image of the left thigh, showing a well-circumscribed, solid subcutaneous mass measuring 7.0 × 5.5 × 4.0 cm with homogeneous signal intensity and no invasion into adjacent structures. (**a**) Coronal view; (**b**) axial view.

Magnetic resonance imaging (MRI) demonstrated a well-circumscribed, solid mass in the subcutaneous tissue of the left thigh, measuring 7.0 × 5.5 × 4.0 cm, with homogeneous signal intensity and no infiltration of adjacent structures (Figure 1). The imaging characteristics suggested a possible soft tissue neoplasm, and low-grade sarcoma was included in the clinical differential diagnosis.

**Figure 2:**
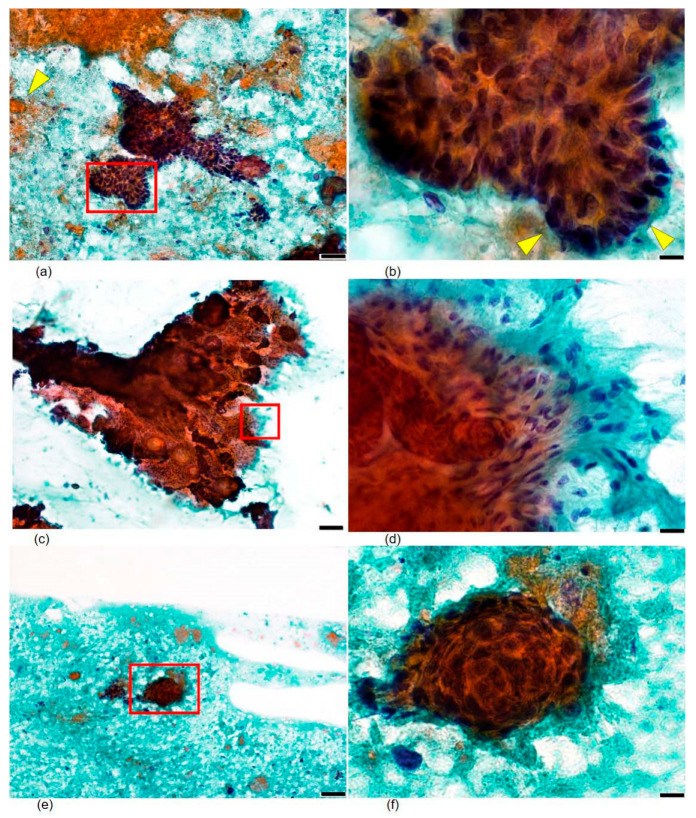
Fine-needle aspiration cytology findings (Papanicolaou stain). (**a**,**b**) Cohesive clusters of basaloid cells with peripheral palisading at ×100 and ×200 magnification, respectively. Orangeophilic keratinous material is visible in the background (yellow arrowheads, (**a**)). ×100 scale bar = 100 μm, ×200 scale bar = 50 μm. (**c**,**d**) Basaloid cells with high nuclear-to-cytoplasmic ratios, finely granular chromatin, and inconspicuous nucleoli at ×400 and ×1000 magnification, respectively. Note that nuclear hyperchromasia, pleomorphism, and mitotic figures are absent. ×400 scale bar = 20 μm, ×1000 scale bar = 10 μm. (**e**,**f**) Delicate spindle-shaped follicular stromal cells (highlighted, red box) at ×200 and ×1000 magnification, respectively. These cells were intimately associated with epithelial cell clusters, forming a characteristic dual-population pattern. ×200 scale bar = 50 μm, ×1000 scale bar = 10 μm.

FNA cytology was performed using a 23-gauge needle under ultrasound guidance, with immediate on-site evaluation. Smears were prepared and stained with Papanicolaou stain. The cytological material was highly cellular and showed the following features (Figure 2):

(1) The background contained scattered amorphous debris and orangeophilic keratinous material with focal calcified deposits, without mucinous matrix material.
(2) Small to medium-sized cohesive cell clusters were composed of basaloid cells arranged in a palisaded pattern at the periphery of the nests, closely resembling the peripheral palisading seen in follicular germinative structures.
(3) Individual basaloid cells possessed round-to-oval nuclei with high nuclear-to-cytoplasmic ratios, finely granular evenly distributed chromatin, and inconspicuous nucleoli. Crucially, nuclear hyperchromasia, significant nuclear pleomorphism, and mitotic figures were absent.
(4) Delicate spindle-shaped follicular stromal cells were present, intimately interspersed among and at the periphery of the epithelial cell clusters, forming a dual-population pattern characteristic of follicular neoplasms. These stromal cells had bland, elongated nuclei and scant cytoplasm and differed clearly from the epithelial basaloid cells.

Integration of these cytomorphological features—palisaded basaloid cell clusters, intimate admixture of follicular stromal cells, keratinous material, and absence of atypia or mucinous stroma—led to a preoperative cytological suggestion of a benign follicular germinative tumor, most consistent with trichoblastoma. Importantly, the absence of mucinous stroma and significant nuclear atypia in the aspirate already argued against basal cell carcinoma at this preoperative stage. Conservative surgical excision was recommended.

**Figure 3:**
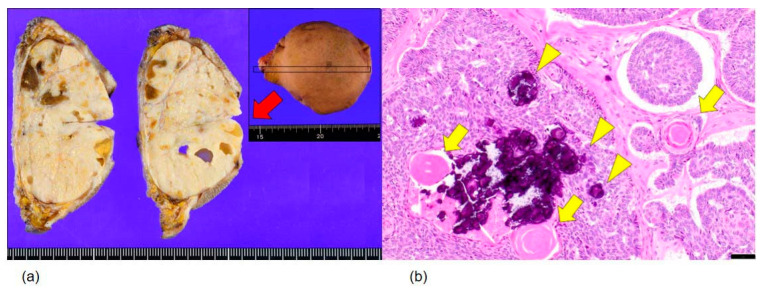
Gross and histopathological findings. (**a**) Macroscopic appearance: encapsulated, solid, grayish–white tumor with a homogeneous cut surface, with no hemorrhage or necrosis. (**b**) Histological section (hematoxylin-eosin staining) demonstrates a well-delineated neoplastic lesion characterized by nests of cells with high nuclear-to-cytoplasmic ratios arranged in a palisading pattern, surrounded by specialized follicular mesenchyme comprising delicate collagen fibers. Some tumor nests contained keratinous cysts (arrows) and calcifications (arrowheads). The histological features corresponded well with the cytological findings, particularly the presence of distinctive stromal components and cohesive basaloid cells. ×100 scale bar = 100 μm.

Grossly, the excised specimen comprised an encapsulated, well-demarcated mass measuring 7.0 × 5.5 × 4.0 cm, with a solid, grayish-white, homogeneous cut surface and no hemorrhage or necrosis (Figure 3a).

Histopathological examination showed a well-delineated tumor composed of basaloid epithelial nests of varying size, with prominent peripheral palisading and intimate association with specialized follicular mesenchyme throughout the tumor (Figure 3b). The stromal component consisted of delicate spindle cells surrounding and supporting the epithelial nests in a pattern characteristic of follicular differentiation. No significant nuclear pleomorphism, increased mitotic activity, necrosis, or perineural or vascular invasion was identified. Surgical margins were free of tumor.

**Figure 4:**
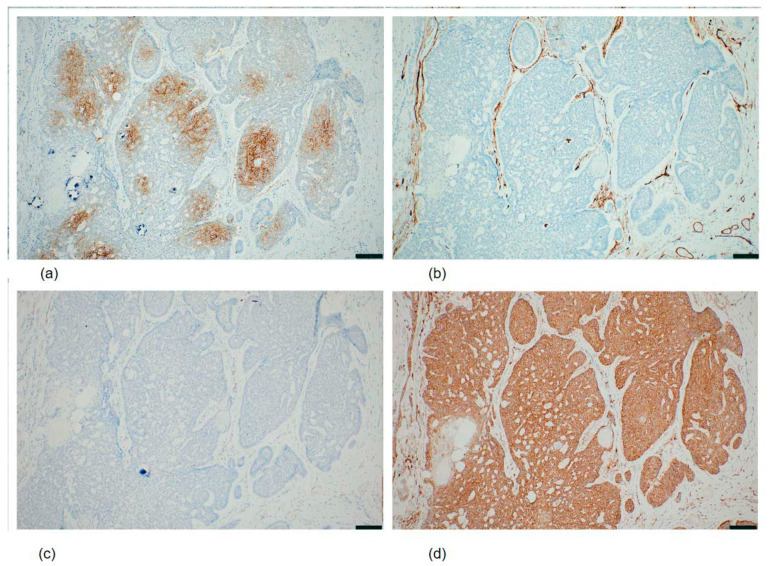
Immunohistochemical findings of the resected specimen (all panels: original magnification ×100). (**a**) BerEP4 (clone Ber-EP4; Roche, ready to use): focal positivity in a subset of tumor cell nests, with other nests remaining negative, consistent with the partial BerEP4 expression characteristic of trichoblastoma. (**b**) CD34 (clone QBEnd/10; Roche, ready to use): diffuse positivity in the follicular stromal component surrounding and separating the basaloid epithelial nests; tumor cells were negative. (**c**) Androgen receptor (clone SP107; Roche, ready to use): complete absence of nuclear staining in tumor cells throughout the sampled area. No internal positive control structures were present in this field; androgen receptor negativity was confirmed in multiple representative sections. (**d**) bcl-2 (clone SP66; Roche, ready to use): positivity in basaloid tumor cells. Collectively, focal BerEP4 expression, CD34-positive follicular stroma, androgen receptor negativity, and bcl-2 positivity supported the diagnosis of trichoblastoma and distinguished this tumor from basal cell carcinoma. Scale bar = 100 μm (all panels).

Immunohistochemistry was performed on formalin-fixed, paraffin-embedded sections of the resected specimen; results are summarized in Table 1. Tumor cells were focally positive for BerEP4 (Ber-EP4; Figure 4a), D2-40 (podoplanin), CD10 (SP67), and c-kit (CD117/Ep10); positive for bcl-2 (SP66; Figure 4d), with expression accentuated in the basaloid cell nests; and negative for CK20 (SP33) and androgen receptor (SP107; Figure 4c). The stroma showed diffuse CD34 positivity (QBEnd/10; Figure 4b) with additional focal CD10 staining. Collectively, the cytomorphological, histopathological, and immunohistochemical findings supported the diagnosis of trichoblastoma.

The patient remained recurrence-free at the 12-month postoperative follow-up, with no clinical or imaging evidence of local recurrence.

## 3. Discussion

The present case illustrates the value of preoperative FNA cytology for accurately characterizing trichoblastoma when it presents at an atypical site. In this patient, initial clinical and MRI findings suggested a low-grade soft tissue malignancy; however, careful cytomorphological analysis resolved the diagnosis prior to surgery. Four key FNA features were decisive for diagnosis: (1) cohesive basaloid cell clusters with peripheral palisading; (2) intimate admixture of delicate spindle-shaped follicular stromal cells forming a recognizable dual population; (3) orangeophilic keratinous material in the background; and (4) an absence of nuclear atypia, mitotic figures, and mucinous stromal material. These features collectively indicated a benign follicular germinative tumor and directed the surgical team toward conservative excision rather than wide resection, avoiding unnecessary morbidity [4,5].

Diagnoses of trichoblastoma by FNA cytology are exceptionally rare in the literature. This reflects both the overall rarity of the tumor and the fact that the clinical and dermoscopic presentation at canonical head and neck locations is often sufficiently characteristic to justify direct excision without prior cytological sampling. Previously, the most direct cytological characterization of trichoblastoma in FNA specimens was provided by Dubb et al. [6], who described the FNA findings of a trichoblastic fibroma, a recognized variant within the trichoblastoma family, in a 58-year-old male presenting with a mass on the thigh, a clinical scenario that closely parallels the present case. These authors noted that the cytomorphological pattern on aspiration resembled that of a cellular fibroadenoma or phyllodes tumor, a feature not previously described in the literature at that time. Peripheral palisading of nuclei at the edges of basaloid cell sheets and squamous eddy formation were identified as diagnostic clues, though potentially focal and easily overlooked. Crucially, even when the tumor occurs in proximity to the breast region, distinction from a fibroadenoma may be difficult if these additional features are not prominent [6], underscoring the importance of meticulous systematic examination of all cytological material obtained from unusual subcutaneous masses. This observation confirms that the distinctive cytological features of trichoblastoma are reproducibly identifiable in aspirates from extra-craniofacial sites, provided that the examining cytologist actively considers this diagnostic possibility. Krishnamurthy and Divya [4] similarly described the FNA features of giant solitary trichoepithelioma, a closely related follicular germinative tumor, identifying cohesive basaloid cell clusters, papillary mesenchymal bodies, and intimately associated fibrillary stromal cells as the defining cytological hallmarks; these features substantially overlap with the cytomorphological pattern observed in both the Dubb et al. series and the present case, further supporting the generalizability of these cytological criteria across the spectrum of follicular germinative neoplasms.

Taken together, these reports suggest that the cytological hallmarks of follicular germinative tumors—cohesive basaloid cell clusters with peripheral palisading, intimately associated spindle-shaped follicular stromal cells forming a recognizable dual-population pattern, keratinous material in the background, and the absence of mucinous stroma or significant nuclear atypia—are identifiable in FNA material when the examining cytologist maintains awareness of this diagnostic category. A critical determinant of diagnostic success is the cytologist’s index of suspicion. At conventional head and neck sites, the differential diagnosis of a basaloid subcutaneous nodule routinely includes follicular adnexal tumors. At anatomically atypical sites such as the thigh, however, the clinical and imaging context is overwhelmingly dominated by soft tissue neoplasms, and the possibility of a benign follicular tumor may not arise spontaneously. The cytological similarity of trichoblastoma aspirates to fibroadenoma or phyllodes tumor, as noted by Dubb et al. [6], represents an additional potential source of diagnostic error when the lesion occurs at an unusual site: without prior knowledge that trichoblastoma can arise in the extremities, even an experienced cytologist might interpret the basaloid cell clusters and stromal component as features of a breast-type biphasic neoplasm rather than a follicular adnexal tumor. This diagnostic trap is especially relevant in the present case, where the large size of the mass, its prolonged clinical course of 20 years, and its subcutaneous location in the thigh all contributed to a clinical and radiological impression of a low-grade soft tissue neoplasm. The diagnostic barrier is most effectively overcome through a systematic cytomorphological approach that evaluates not only nuclear features but also the stromal compartment and background material of the aspirate.

In the present case, these two components—basaloid cell clusters with peripheral palisading, and delicate spindle-shaped stromal cells intimately interdigitating with the epithelial nests—formed a recognizable dual-population pattern that, when integrated with the absence of mucinous stroma, the presence of orangeophilic keratinous material, and the lack of significant nuclear atypia, supported a preoperative cytological assessment of a benign follicular germinative tumor. The additional absence of the mucinous stromal matrix, which characteristically accompanies basal cell carcinoma (BCC) aspirates, was particularly helpful in the present context, effectively excluding the most important cytological differential diagnosis. Both follicular adnexal tumors and BCCs yield cohesive basaloid cell clusters with peripheral palisading; thus, cytological distinction can be challenging [7,8]. However, the stromal component in BCC, when aspirated, tends to be less cellular and is often associated with mucinous matrix material. The presence of orangeophilic keratinous debris also supports follicular rather than BCC differentiation, as BCC rarely produces this type of keratinous material [7,8]. Additionally, a complete absence of hyperchromasia and nuclear pleomorphism argues against BCC. Collectively, the present case provides additional evidence that this systematic, feature-by-feature cytological approach is applicable even when the clinical presentation strongly favors a mesenchymal malignancy, and adds to the very limited published literature documenting the preoperative FNA-based characterization of trichoblastoma at extra-craniofacial sites.

The key FNA features described in this case should also prompt the consideration of other basaloid adnexal tumors, including trichoepithelioma and cylindroma. Trichoepithelioma shares follicular differentiation and cytological overlap with trichoblastoma, and distinction on cytology alone may not always be possible. However, as both tumors are benign and managed similarly, this distinction has minimal clinical consequences in the preoperative setting. Cylindroma produces characteristic hyaline droplets and a jigsaw-puzzle growth pattern on histology, which were not present in this case. Although large soft tissue neoplasms, including synovial sarcoma and epithelioid sarcoma, may produce cohesive epithelioid cell clusters on FNA, the bland nuclear morphology and characteristic follicular stromal component observed in the present case argue strongly against a mesenchymal malignancy. In summary, a systematic, feature-by-feature cytological approach remains essential even when the clinical presentation strongly favors a mesenchymal malignancy.

A broad immunohistochemical panel was also applied in the present case given the atypical clinical presentation, which necessitated exclusion of neuroendocrine differentiation among other diagnostic considerations. SSTR2 (somatostatin receptor type 2) was included in the panel specifically to evaluate for possible neuroendocrine differentiation, given that the atypical clinical presentation of a large subcutaneous mass raised a broad differential diagnosis. The observed membranous SSTR2 positivity does not indicate neuroendocrine lineage; rather, SSTR2 expression has been reported in various non-neuroendocrine epithelial tumors, and its significance in trichoblastoma remains to be clarified. The immunohistochemical findings confirmed and extended the cytological diagnosis of trichoblastoma. The partly positive BerEP4, diffusely CD34-positive stroma, and negative androgen receptor and CK20 are consistent with the established immunophenotype of trichoblastoma and support its distinction from BCC [9,10,11]. BCC characteristically demonstrates diffuse BerEP4 positivity and androgen receptor expression, whereas trichoblastoma tends to show focal or variable BerEP4 staining and is androgen receptor-negative [9,10,11]. The diffuse CD34 positivity in the stroma, reflecting the specialized follicular mesenchyme, is another helpful diagnostic feature because BCC stroma typically lacks significant CD34 expression [10]. Finally, focal D2-40 (podoplanin) positivity in tumor cells is consistent with follicular outer root sheath differentiation and has been reported in trichoepithelioma and related tumors; podoplanin expression is significantly less frequent in BCC, supporting its utility in this differential diagnosis [12]. The characteristic immunohistochemical findings—particularly focal BerEP4 expression, diffuse CD34-positive follicular stroma, androgen receptor negativity, and bcl-2 positivity—provided definitive confirmation of the cytological impression and established the diagnosis of trichoblastoma.

Moreover, the present case extends the recognized topographic range of trichoblastoma. Trichoblastoma at anatomical sites beyond the conventional head and neck region is exceptionally uncommon. Among extra-craniofacial locations, the lower extremities have been documented very rarely. Frings et al. [13] reported a case of giant trichoblastoma involving the leg, and emphasized the diagnostic challenge posed by the large size and unusual location of the lesion, which initially raised clinical concern for a malignant process. Nguyen et al. [14] described an atypical giant trichoblastoma with an unusual clinical presentation, further illustrating the morphological and topographic heterogeneity that can characterize trichoblastoma outside its canonical distribution, as well as the importance of histopathological confirmation in atypical cases. Shimazaki et al. [15] reported trichoblastoma of the skin occurring in the breast, demonstrating that follicular germinative tumors can arise in soft tissue remote from the classical sun-exposed craniofacial regions. The present case, involving the left thigh of a 49-year-old male who had harbored the lesion for approximately 20 years before seeking evaluation, adds to this body of evidence and further extends the recognized topographic range of trichoblastoma. These reports also highlight the problem of delayed or misattributed diagnoses for sites where clinicians and radiologists do not routinely consider adnexal tumors. Specifically, incorrect diagnoses of malignant mesenchymal neoplasm have significant implications for the invasiveness of subsequent surgical management. In the absence of cytological data, the initial MRI findings in the present case, which suggested a possible low-grade soft tissue sarcoma, would typically prompt a management algorithm including wide surgical excision with clear margins, preoperative multidisciplinary oncological review, and in some institutional settings, consideration of neoadjuvant therapy or percutaneous core needle biopsy as a preliminary diagnostic step. These interventions are associated with substantial morbidity in the thigh, where wide resection may necessitate extensive sacrifice of surrounding musculature and neurovascular structures, potentially resulting in lasting functional impairment, chronic pain, and the need for complex reconstructive procedures. The preoperative cytological assessment of a benign follicular germinative tumor fundamentally redirected surgical planning in the present case, enabling conservative local excision and avoiding unwanted consequences. This direct impact on surgical decision-making represents the most clinically meaningful contribution of preoperative FNA cytological assessment. Awareness of the capacity of trichoblastoma to present at anatomically remote sites, combined with systematic preoperative cytological evaluation when clinically feasible, represents the most effective strategy for preventing diagnostic pitfalls and associated surgical consequences in the multidisciplinary management of soft tissue masses at atypical anatomical locations.

Even at conventional anatomical sites, trichoblastoma is susceptible to misdiagnosis through partial tissue sampling. Demant et al. [3] described a case of giant trichoblastoma of the forehead in which initial punch biopsy yielded a diagnosis of BCC, with the correct diagnosis established only from the definitive surgical excision specimen. Thus, the defining architectural features of trichoblastoma, including the characteristic follicular stromal component and specific spatial relationship between epithelial nests and stromal cells, may be inadequately represented in a limited biopsy specimen. In the context of atypical anatomical sites, this sampling challenge is compounded by low preoperative clinical suspicion, creating a setting in which even adequate tissue sampling may not yield the correct diagnosis if the pathologist does not consider follicular adnexal neoplasia in the differential diagnosis. In such a scenario, preoperative FNA cytology that explicitly considers both epithelial and stromal components of the aspirate represents a critical diagnostic pivot preventing unnecessary surgery with potential morbidity. The present case illustrates that approaching FNA cytological evaluation with awareness of the defining features of trichoblastoma can lead to the correct preoperative diagnosis, even for highly atypical anatomical sites. From a practical perspective, this cytological approach requires no specialized equipment and is readily applicable in any cytopathology laboratory with experience in FNA interpretation. Finally, preoperative cytological characterization, when accurate, has direct therapeutic consequences by allowing appropriate tailoring of surgical management. Incorporating this diagnostic framework into routine practice for subcutaneous masses at unusual sites may help reduce diagnostic delays and prevent unnecessary surgical morbidity in patients presenting with trichoblastoma at extra-craniofacial locations.

Several limitations inherent to this single case report warrant acknowledgment. First, the cytomorphological observations described here are based on a single FNA specimen from a large nodular variant of trichoblastoma, and their reproducibility across other histological subtypes, smaller lesions, or FNA specimens with suboptimal cellularity cannot be reliably determined from this case alone. Dedicated prospective studies incorporating larger numbers of cases would be required to define the sensitivity and specificity of the cytological criteria described here and to establish their reliability across the full histological spectrum of trichoblastoma. Second, molecular analysis—including mutational profiling or immunohistochemical assessment of follicular stem cell markers that have been reported to differentiate trichoblastoma from BCC—was not performed on the resected specimen in the present case; such studies could provide additional insights into the molecular relationship between trichoblastoma and other follicular adnexal neoplasms and could potentially support preoperative cytological diagnosis if applied to cell block material. Third, the postoperative follow-up period of 12 months, while demonstrating the absence of local recurrence, is insufficient to fully characterize the long-term clinical behavior. Trichoblastoma is regarded as a benign neoplasm with a low risk of recurrence after complete excision [1]; however, rare cases of malignant transformation to trichoblastic carcinoma have been documented [1], and continued clinical surveillance remains advisable. Fourth, immunohistochemical analysis in the present case was performed exclusively on sections of the formalin-fixed, paraffin-embedded resected specimen; incorporation of immunocytochemistry into the preoperative cytological workup, using cell block material prepared from the FNA aspirate, could potentially further refine the preoperative characterization of this rare entity and provide an additional diagnostic tier beyond cytomorphological assessment alone. Future case reports incorporating cell block immunocytochemistry, as well as prospective studies exploring the utility of rapid on-site evaluation in trichoblastoma aspirates, would contribute meaningfully to the currently limited literature on the preoperative cytological diagnosis of trichoblastoma at extra-craniofacial sites. Despite these limitations, the present case demonstrates that careful cytomorphological analysis, integrating evaluation of both the epithelial and stromal compartments of the aspirate with systematic exclusion of features characteristic of BCC and soft tissue sarcomas, can yield a clinically actionable preoperative cytological characterization of trichoblastoma even at anatomically atypical locations. This finding supports the incorporation of FNA cytology into the diagnostic algorithm for subcutaneous masses at unusual sites and underscores the importance of maintaining a broad cytological differential diagnosis that includes rare benign adnexal tumors alongside the more commonly encountered soft tissue neoplasms.

## 4. Conclusions

In this case, FNA cytology successfully characterized trichoblastoma at an unusual anatomical site. Four key cytomorphological features, cohesive basaloid cell clusters with peripheral palisading, intimate admixture of delicate follicular stromal cells, orangeophilic keratinous material, and an absence of significant nuclear atypia, enabled the correct preoperative diagnosis of trichoblastoma. This cytological characterization guided conservative surgical management, thereby avoiding unnecessary wide resection. Immunohistochemistry confirmed the diagnosis and supported the distinction from BCC. Careful attention to cytomorphological detail, particularly the dual epithelial–stromal cell population characteristic of follicular germinative differentiation, is crucial for the accurate recognition of trichoblastoma at atypical locations on FNA cytology.

## Figures and Tables

**Table 1:** Immunohistochemical findings.

Antibody	Clone	Manufacturer	Dilution	Result
SSTR2	UMB1	Abcam	1:100	Positive (membranous)
bcl-2	SP66	Roche	RTU	Positive
BerEP4	Ber-EP4	Roche	RTU	Focally positive
D2-40 (podoplanin)	D2-40	Roche	RTU	Focally positive
CD10	SP67	Roche	RTU	Focally positive (tumor cells & stroma)
c-kit (CD117)	Ep10	Santa Cruz	1:100	Focally positive
CD34	QBEnd/10	Roche	RTU	Positive (stroma, diffuse)
CK20	SP33	Roche	RTU	Negative
Androgen receptor	SP107	Roche	RTU	Negative

Abbreviations: RTU, ready to use. Result categories: Positive = diffuse staining (>50% of tumor cells); Focally positive = 10–50% of tumor cells or focal stromal staining as specified; Negative = <10% of tumor cells.

## Data Availability

The data supporting the findings of this study are available from the corresponding author upon reasonable request.

## Abbreviations

The following abbreviations are used in this manuscript:

FNA	Fine-needle aspiration
MRI	Magnetic resonance imaging
BCC	Basal cell carcinoma

## References

[1] Requena L., Calonje J.E., Kazakov D.V., Kolm I., WHO Classification of Tumours Editorial Board (2025). Trichoblastoma. WHO Classification of Skin Tumours.

[2] Headington J.T. (1976). Tumors of the hair follicle. A review. Am. J. Pathol..

[3] Demant M., Saltvig I., Trøstrup H., Schmidt V.J., Hesselfeldt J. (2020). Don’t judge a tumor by its biopsy!. Case Rep. Dermatol..

[4] Krishnamurthy J., Divya K. (2010). The cytology of giant solitary trichoepithelioma. J. Cytol..

[5] Headington J.T. (1970). Differentiating neoplasms of hair germ. J. Clin. Pathol..

[6] Dubb M., Michelow P., Grayson W. (2009). Cytologic features of trichoblastoma in fine needle aspiration biopsies. Acta Cytol..

[7] Stanoszek L.M., Wang G.Y., Harms P.W. (2017). Histologic mimics of basal cell carcinoma. Arch. Pathol. Lab. Med..

[8] Patel P., Nawrocki S., Hinther K., Khachemoune A. (2020). Trichoblastomas mimicking basal cell carcinoma: The importance of identification and differentiation. Cureus.

[9] Swanson P.E., Fitzpatrick M.M., Ritter J.H., Glusac E.J., Wick M.R. (1998). Immunohistologic differential diagnosis of basal cell carcinoma, squamous cell carcinoma, and trichoepithelioma in small cutaneous biopsy specimens. J. Cutan. Pathol..

[10] Turnbull N., Ghumra W., Mudaliar V., Vella J., Sanders D.S.A., Taibjee S., Carr R. (2018). CD34 and BerEP4 are helpful to distinguish basaloid tricholemmoma from basal cell carcinoma. Am. J. Dermatopathol..

[11] Bourlond F., Velter C., Cribier B. (2021). Androgen receptor expression in epidermal and adnexal tumours. Ann. Dermatol. Venereol..

[12] Plaza J.A., Ortega P.F., Bengana C., Stockman D.L., Suster S. (2010). Immunolabeling pattern of podoplanin (D2-40) may distinguish basal cell carcinomas from trichoepitheliomas: A clinicopathologic and immunohistochemical study of 49 cases. Am. J. Dermatopathol..

[13] Frings V.G., Goebeler M., Kneitz H. (2017). Dermpath & Clinic: Giant trichoblastoma of the leg. Eur. J. Dermatol..

[14] Nguyen L.V., Masouminia M., Choy J.O., Peng S.K., Ji P., French S.W. (2017). Atypical giant trichoblastoma: An unusual presentation. Exp. Mol. Pathol..

[15] Shimazaki H., Anzai M., Shinsuke A., Endo H., Kato K., Yamasaki T., Tamaki K., Tamai S. (2001). Trichoblastoma of the skin occurring in the breast: A case report. Acta Cytol..

